# Supplementary material for: Isolation of Epizootic Hemorrhagic Disease Virus Serotype 10 from Culicoides tainanus and Associated Infections in Livestock in Yunnan, China
Source: Viruses. 2024 Jan 24;16(2):175. doi: 10.3390/v16020175 (PMC10892452; doi:10.3390/v16020175)
Supplement: Supplementary file 1 [file viruses-16-00175-s001.zip › viruses-2788456-supplementary.pdf]

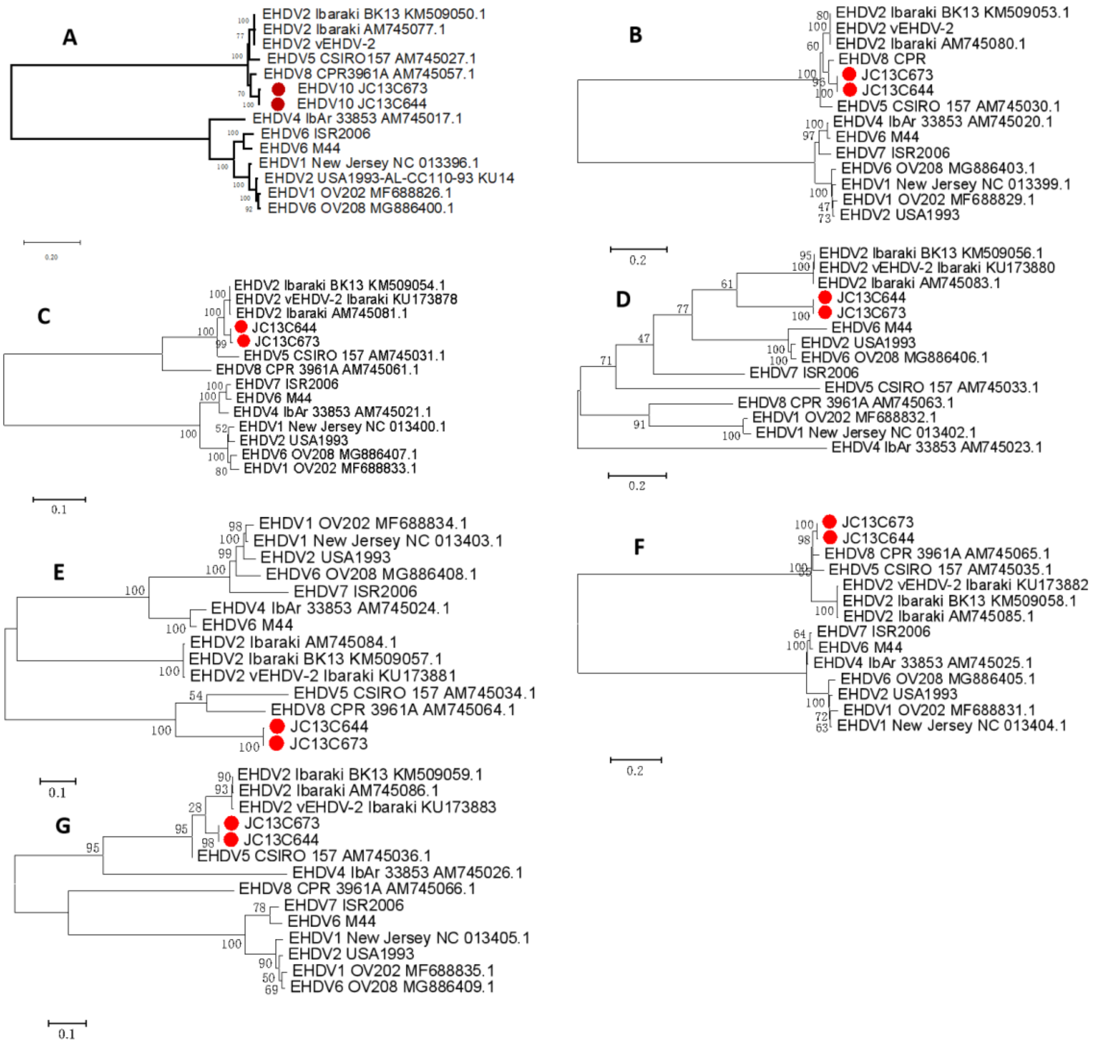

Figure S1. ML phylogenetic trees of Seg-1 (A), Seg-4(B), Seg-5(C), Seg-7(D), Seg-8(E), Seg-9(F) and Seg-10(G) of JC13C673 and JC13C644 strains EHDV isolated from the border area shared by China, Laos, and Vietnam. The best substitution model is TN93+G+I(Seg-1), TN93+I(Seg-4), TN+I(Seg-5), GTR+G+I(Seg-7), HKY+G+I(Seg-8), TN93+I(Seg-9) and HKY+G(Seg-10); The bootstrap value is 1000.

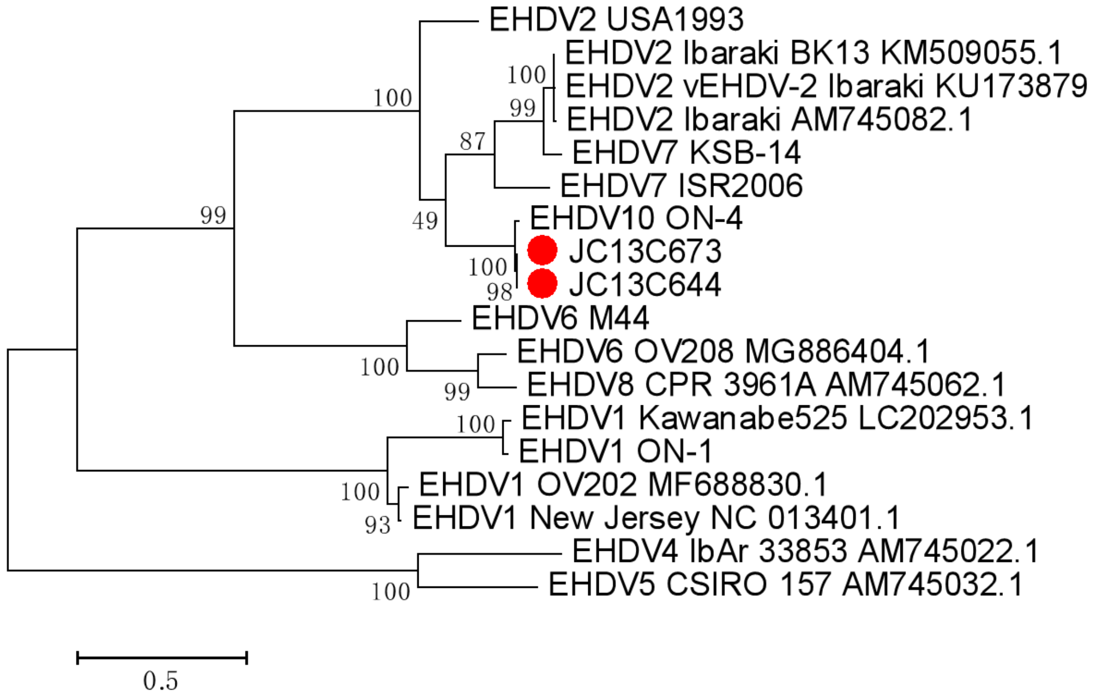

Figure S2. ML phylogenetic trees of Seg-6 of JC13C673 and JC13C644 strains EHDV isolated from the border area shared by China, Laos, and Vietnam. The best substitution model is GTR+G(Seg-6); The bootstrap value is 1000.

Table S1. The sequence information used in this study.

NO.	strain	location	host	date	LOCUS	Serotype
1	Kawanabe525	Japan:Kagoshima(JP)	Bos taurus	1985	LC202942, LC202942, LC202953	EHDV1
2	ON-1/E/13	Japan:Okinawa(JP)	Bos taurus	2013	LC202950, LC202972, LC202961	EHDV1
3	OV202	USA	Odocoileus virginianus	2015	MF688826-MF688835	EHDV1
4	USA1955/01	USA:New Jersey(NJ)	unk	1955	NC_013397-NC_013405	EHDV1
5	6010	France: Reunion Island	cattle	unk	JX965387.1（VP2）	EHDV1
	Ibaraki	Japan(JP)	Cattle	1959	AM745077.1-AM745086.1	EHDV2
6	Ibaraki BK13	Japan(JP)	cattle	1997	KM509050.1-KM509059.1	EHDV2
7	4976	France: Guadeloupe Island(FR)	cattle	2011	JX965386.1（VP2）	EHDV2
8	vEHDV-2 Ibaraki	Italy	unk	2011	KU173874.1-KU173883.1	EHDV2
9	CC110-93	USA: Alabama(AL)	unk	1993	KU140734.1（VP2）	EHDV2
10	IbAr 33853	Nigeria	Culicoides spp.	1968	AM745017- AM745026	EHDV4
11	CSIRO 157	Australia	cattle	1977	AM745027.1- AM745036.1	EHDV5
12	M44/96	South Africa	Bos taurus (cattle)	1996	HM636907.1-HM636916.1	EHDV6
13	OV208	USA	Odocoileus virginianus	2016	MG886400.1- MG886409.1	EHDV6
14	Guadeloupe/5124	France: Guadeloupe Island	bovine	2010	HQ848380.1（VP2）	EHDV6
15	HG-1/E/15	Japan:Hyogo(JP)	Bos taurus	2015	LC320035.1（VP2）, LC320035.1（VP3）	EHDV6
16	ISR2006/02	Israel: Jordan Valley	cattle	2006	KM391743, KM391727, KM391737, KM391751, KM391725, KM391753, KM391739, KM391745, KM391736, KM391732	EHDV7
17	KSB-14/E/97	Japan:Kagoshima(JP)	Bos taurus	1997	LC202943.1（VP2）, LC202954.1（VP3）, LC202965.1（VP5）	EHDV7
18	YN09-04	China:Yunnan	Cattle	2013	MK656453.1-MK656462.1	EHDV7
19	CPR 3961A	Australia	Cattle	1982	AM745057-AM745066	EHDV8
20	ON-4/B/98	Japan:Okinawa(JP)	Bos taurus	1998	LC202944.1（VP2）, LC202955.1（VP3）, LC202966.1（VP5）	EHDV10
21	JC13C673	China:Yunnan(YN)	Culicoides	2013	MT013314-MT013323	EHDV10
22	JC13C644	China:Yunnan(YN)	Culicoides	2013	MT013324-MT013333	EHDV10

Note：unk refers to the information is unknown
